# Interventional Cultural and Language Assistance Program: Associations between Cultural and Linguistic Factors and Satisfaction with Cancer Care

**DOI:** 10.4172/2161-0711.1000503

**Published:** 2017-02-20

**Authors:** R Costas-Muniz, J Amir, M Paris, D Spratt, J Arevalo-Perez, S Fareedy, CJ González, F Gany, M Camacho-Rivera, JR Osborne

**Affiliations:** Department of Psychiatry, Weill Cornell Medical College, New York, USA

**Keywords:** Limited english proficiency, Satisfaction with care, Immigrant health, Patient access

## Abstract

Addressing language and cultural nuance is required to improve the quality of care among all patients. The tenth version of the National Standards for Culturally and Linguistically Appropriate Services in Health Care (CLAS) recommends implementing ongoing assessments to integrate specific actions into measurement and continuous quality improvement activities. To this end, we have created the Interventional Cultural and Language Assistance Program (ICLAP). As part of ICLAP, we conducted a cross-sectional needs assessment survey with 564 consecutive patients receiving outpatient Positron emission tomography-computed tomography (PET/CT) imaging at a comprehensive cancer center in the five most prevalent languages of New York City: English, Spanish, Russian, Chinese, and Arabic. The purpose of this study is to describe the language assistance characteristics and needs of a sample of patients receiving care in the cancer center. We examined the relationship between race, ethnicity, birthplace, communication and language assistance characteristics and the satisfaction with the care received. Our results show that race and ethnicity, birthplace, cultural beliefs, language assistance, and communication characteristics were all factors associated with patients’ satisfaction with care, illustrating that there is an unmet need among cancer patients to have cultural and linguistic sensitive services.

## Introduction

The ability to provide patient-centered cancer care to all patients is intimately tied to linguistically and culturally competent communication [1,2]. The Nuclear Medicine (NM) community, however, has not historically considered communication a centerpiece of its delivery model. We assume this is a historical relic of the creation of the specialty. Three decades ago, patient interactions in NM were dominated by radioiodine therapy of thyroid cancer in conjunction with endocrinologists. The majority of the other NM procedures over the next several decades were considered diagnostic imaging and communication was generally delegated to the referring physician. The complexity of NM studies, and the need for communication skill has grown considerably, however, as evidenced by the large number of cancer patients being diagnosed and followed with Positron Emission Tomography (PET/CT) in the outpatient setting. Yet, best practices for interacting with patients being evaluated with advanced diagnostics has not grown to match the pace of the increased time and complexity of the services rendered. It is certainly appropriate that the Nuclear Medicine community rethink its historic precedent with regard to communication.

U.S. Census bureau statistics also make this an updated NM approach a necessity. Approximately 24 million (8.6%) of the U.S. population and 1.8 million people in New York City have limited English proficiency (LEP) [3], a growth of almost 50% over the past decade, with nearly half (49%) speaking a language other than English at home [3]. Language and cultural barriers compromise patients’ ability to obtain and to comprehend their health information [4]. For example, Spanish speakers have less favorable experiences with provider communication and perceive office staff as less helpful than their English-speaking counterparts [5]. Language concordance and quality of interpretation in the clinical encounter may impact patients’ perception of patient-provider communication [2]. Immigrants with limited English proficiency face additional barriers like receiving limited counseling from their physicians [6] and feeling less satisfied with their medical decisions for their treatment [7]. Previous studies have also revealed racial and ethnic disparities in patient-provider communication and quality of care. Palmer et al. reported that Asian cancer survivors report poorer follow-up care communication and care quality [8] and Pippins, et al. [6] found that Latino patients with limited English language proficiency have more negative experiences of primary care than their English language proficient counterparts, with decreased access (longer wait times and greater difficulty obtaining information or advice) and less continuity of services. Racial and ethnic minority patients often have less access to medical information and face challenges making medical decisions due to socioeconomic, cultural, and language factors and their communication with health care providers [3]. Ethnic minority individual often receive less biomedical information during the clinical encounter and receive patient-centered information [5].

It is uncertain if awareness of interpreter services, need for these services, and use of interpretation services has an impact on patients’ satisfaction with care in the context of this study centered in an NM outpatient practice. Therefore, we first performed an initial cross-sectional survey-based needs assessment so that a follow-up intervention could have a more data-centric design. As a first step toward measuring cultural and communication factors in an outpatient imaging setting, we surveyed over a 3 month period patients scheduled to have a PET/CT at our outpatient imaging setting at a comprehensive cancer center to determine the impact of demographic characteristics, communication and language assistance needs and utilization, and satisfaction with care.

## Methods

### Participants and procedure

A cross-sectional tablet-based needs assessment survey was conducted with 564 cancer patients waiting for an outpatient imaging procedure between January 2016 to May 2016, as part of the Interventional Cultural and Language Assistance Program (ICLAP) at our comprehensive cancer center. The recruitment period for the study was between January 2016 to May 2016. In order to be eligible for the study, patients had to be scheduled for an outpatient clinical F-Fluorodeoxyglucose (FDG) PET/CT scan. Potential participants were informed that the study was voluntary, anonymous and confidential. After obtaining informed consent, Session Assistants provided a tablet to patients to self-administer the survey, which was available in five of the most widely spoken languages in New York City: English, Spanish, Russian, Chinese, and Arabic. Session Assistants invited 575 consecutive patients to participate, eleven could not complete the survey because they speak other languages not available in the survey (i.e. Japanese, Hebrew, Polish). Surveys for 564 outpatients were considered evaluable.

### Measures

All measures were assessed by self-report. The tablet-based assessment survey included four sections: sociodemographic, cultural, migration, linguistic and cultural beliefs questions, need and use of interpretation services, and satisfaction with care. The sociodemographic section included age, gender, marital status, employment status, education, income level. The cultural and migration-related sections included questions assessing participants’ preferred and dominant language, race, ethnicity, country of origin and a question assessing if their cultural background influences their medical decisions. The interpretation needs section assessed need for interpretation in medical settings, preferred language for healthcare, use of interpretation services in the clinic and frequency of use of such services. Satisfaction with the outpatient imaging clinic, satisfaction with the cancer center, and satisfaction with the interpretation services were assessed using a 1–10 rating scale.

### Analysis

Statistical analyses were conducted using the SPSS19 software package. First, the demographic characteristics of the 564 participants were described using descriptive statistics. Then, the need for and use of interpretation services questions were described. Bivariate logistic regression models were used to identify linguistic and cultural factors significantly associated with satisfaction with clinical care at the service level and at the institution level. Satisfaction with care (with clinic and institutional care) was assessed with a question with a response format of a scale from 1–10; given that the scale was severely skewed, the scales were reduced to binomial outcomes for the purpose of analysis (1–7 lower satisfaction, 8–10 higher satisfaction).

Potential explanatory variables included sociodemographics (age, gender, employment status, and education level), linguistic and migration-related factors (race, ethnicity, dominant and preferred language, English proficiency birthplace, linguistic comprehension during clinical encounters), interpretation-related (awareness, use, and need for interpretation, type of interpreter, and satisfaction with interpretation services), and beliefs influencing medical decisions (religious, cultural, and sexual orientation). Unadjusted odds ratios (ORs) and 95% confidence intervals were calculated to assess the relationship of these factors with satisfaction with nuclear medicine clinical care and with clinical care provided by the cancer center.

Logistic regression models were adjusted for relevant variables (age, gender, employment status, education level, and diagnosis) to determine the contribution of linguistic, migration-related, interpretation-related factors and beliefs influencing medical decisions to satisfaction with care. A two-sided p significant level of less than 0.05 was considered statistically significant.

## Results

### Participants

Five hundred seventy participants were approached; eleven could not complete the survey because the survey was unavailable in their preferred languages (Japanese, Hebrew, Polish). Surveys for 564 outpatients were considered evaluable. The mean age of the sample was 52 years (SD=18.1) and nearly 57% of the respondents were male. With regards to race and ethnicity, 14% of the sample was Hispanic or Latino and 8% was Black or African-American. Three quarters of the sample were born in United States, almost 6% were born in Europe, 4% in Eastern Asia and the Philippines, and 4% in Latin America and the Hispanic Caribbean. The most common diagnosis was lymphatic cancer (18%), followed by lung cancer (12%) (Table 1).

The majority of the sample reported that they prefer English for their healthcare services (94%). Almost two thirds of the sample (62%) were aware of interpretation services, 6% reported that they needed an interpreter and 5% that they received interpretation in the clinic. However, 11% reported that their dominant language was a non-English language. Patients more frequently reported using ad-hoc interpretation, family members and/or friends interpreted for them when needed.

One out of six patients (17%) responded that they did not understand their doctor because of language; 12% of patients responded that they would prefer to speak about their medical condition in a language other than English; 12% of the respondents that they did not fully understand what the doctor said; and 18% of the respondents thought that their doctor did not understand them (Table 2).

In analyses adjusting for demographic characteristics (age, gender, education, employment status) several cultural, language assistance, and communication factors were significant predictors of less satisfaction with imaging clinic and institutional care (Table 3). Ethnicity and country of birth were significant predictors of satisfaction with care. East Asian patients and patients born in East Asia or the Philippines were less satisfied with the care received at the cancer comprehensive center than non-Hispanic White patients and patients born in the US (OR=2.95, CI=1.18–7.38; OR=4.69, CI=1.72–12.84, respectively). However, Latino or Hispanic patients were more likely to be satisfied with the care received at the cancer center than non-Hispanic White patients (OR=0.09, CI=10.01–0.69). Further, patients who stated that their ethnic and/or cultural background affect their medical care decisions were two times more likely to be unsatisfied with the care provided by the service and by the cancer center than their counterparts (OR=2.63, CI=1.37–5.05; OR=2.24, CI=1.015.06, respectively).

Patients who reported preferring a non-English language for their healthcare were three times more likely (OR=3.49, CI=1.35–8.98) to be less satisfied with the care at the service and eight times more likely to be dissatisfied with the care received at the cancer center (OR=8.44, CI=3.02–23.57). Patients who needed interpretation were three times more likely to be dissatisfied with the care of the service (OR=3.03, CI=1.27–7.26) and five times less likely to be satisfied with the care of the center (OR=5.49, CI=2.11–14.30). Patients who used interpretation in the clinic were three times less likely to be satisfied with the care of the center (OR=3.01, CI=1.53–5.92).

Patients who reported that they do not understand their doctor because of language were almost two times more likely to be dissatisfied with the care of the service (OR=1.86, CI=1.05–3.29) and three times less likely to be satisfied with the care of the center (OR=3.01, CI=1.53–5.92). Patients who reported feeling more comfortable discussing their medical condition in a language other than English were two times more likely to be less satisfied with the service (OR=2.18, CI=1.15–4.11) and three times less likely to be satisfied with the care of the center (OR=3.68, CI=1.78–7.61). Further, patients who reported that they did not fully understand what the doctor said were four times more likely to be less satisfied with the care of the service (OR=4.40, CI=2.43–7.94) and nine times less likely to be satisfied with the care of the center (OR=9.05, CI=4.52–18.11).

## Discussion

This study was designed to explore patient perception of quality of care received during an outpatient imaging encounter. While the overall satisfaction with care at our comprehensive cancer center is high, we still perceived the need to explore care delivery in the context of race, ethnicity, birthplace, language preference and the presence of interpreter. In this first study we found that these factors (race and ethnicity, birthplace, cultural beliefs, and language assistance) are associated with patients’ satisfaction metrics. This is the first study to measure cultural and linguistically competent delivery of care in a Radiology/Nuclear Medicine setting. The diagnostic and therapeutic branch of Nuclear Medicine has grown considerably, and now involves having critical interactions with many cancer patients being diagnosed and followed with PET/CT. Our findings demonstrate a clear unmet need to have services provided in these encounters with greater cultural and linguistic competence.

Our findings show that Asian patients were less satisfied with care, but Latino patients reported being more satisfied with the care received. This finding is consistent with the study of Palmer and colleagues [8] who found that Asian cancer survivors report worse communication, quality care, and self-efficacy compared with White survivors. In contrast, in this study Latino patients were more likely to be satisfied with their care [8]. More research with larger samples of Asian and Latino samples is necessary to elucidate what are the key factors that influence satisfaction with care among Latino and Asian patient populations.

Studies have revealed that access to linguistic assistance and culturally competent care are critical for optimal care of cancer patients [8]. Patients who reported preferring a non-English language for their care, who needed and used interpretation services, and who reported difficulty understanding or communicating with their providers because of language were less satisfied with their care. Excellent patient-clinician communication can have a long-term effect on patients’ long-term outcomes by facilitating or improving access to needed care, increased patient knowledge and shared understanding, enhanced therapeutic alliances (among clinicians, patient, and family), enhanced self-management, activating social support and advocacy resources, increasing the quality of medical decisions (e.g., informed, clinically sound, concordant with patient values, and mutually endorsed), and enabling patient agency (self-efficacy and empowerment) [9]. However, if patients and clinicians fail to communicate due to language discordance and lack of language assistance, it can have devastating consequences for the care of patients. Clinicians can also improve their communication and culturally competent care with LEP and ethnic minority patients by referring them to care managers to assist them with their communication needs. Care managers worked directly with individual patients, helping them to monitor their health, facilitating the provision of linguistic and culturally competent care, and providing the necessary information and advice to promote patient empowerment, enhance self-management skills, and achieve better compliance with care recommendations [10].

Health care facilities in the United States resort to multiple strategies to address language barriers, and while matching patients with providers who speak their language is the ideal scenario, the use of health care interpreters is by far the most common strategy. Looking nationally at the practice of Nuclear Medicine/Molecular Imaging, the application of linguistic and culturally responsive practices cannot be elicited from a search of the published literature. The current practice of nuclear medicine involves not only the interpretation of diagnostic imaging but also the administration of therapy. This evolution is evident in the name change at many institutions from Nuclear Medicine to Molecular Imaging and Therapy. The imaging acquisition and clinical read component is essentially “colorblind” (physicians complete the analysis without a patient encounter and knowledge of cultural and ethnic affiliation), but the administration of therapy is decidedly not colorblind. In these broadening therapeutic applications, the clinicians in this field and the supporting staff find themselves at a crossroads where meaningful care cannot be delivered without probing how it interfaces with the community of patients. After several decades of treating patients for thyroid cancer with radioiodine, the therapeutic branch of nuclear medicine has grown to other cancers such as lung, neuroendocrine, and breast. Patient provider communication must improve and optimal training must be developed and implemented. Additionally, since most of these treatments require radiation safety precautions, meaningful communication is needed to address safety concerns. At our center all of radionuclide therapy patients receive oral and written radiation safety precautions. Lack of understanding or partial compliance might result in unnecessary exposure to patient, immediate relatives, healthcare personnel or the general public. Often times, detailed information about patients’ living conditions is necessary to provide them with individual precautions. This can only be obtained with optimal communication with the patient. It requires detailed questions and exchanges about sleeping, accommodation or transportation arrangements, to name a few. Therefore, creating a framework for improving cultural and linguistic competency would respond to an unmet need in Molecular Imaging.

Studies providing information about the potential impact of language barriers on quality of care in Nuclear Medicine are warranted. Such studies are needed to develop an evidence base to inform health services delivery and policy. Evidence suggests that cancer disparities in ethnic minorities are related to receipt of lower quality care [11–13]. Thus, understanding how language barriers hinder care represents an important direction in the elimination disparities. Future studies should consider interventions that address the barriers faced by patients with limited English proficiency, including attracting a more culturally and linguistic diverse workforce, improving cultural responsiveness of the medical staff through training and consultations as needed, improving the cultural responsiveness of institutions by improving the integration and availability of linguistic services and the evaluation of the implementation of these services. Further, although addressing language needs and barriers is a federal mandate, standardization and systematization of approaches to ensure linguistic competence in healthcare is needed [14,15]. Future studies should also contribute to the standardization and systematization of the assessment measures of providers’ linguistic competence and the institutional implementation of the federal mandate. Comprehensive assessments, including the patients’ perspective, of the implementation of the mandate are needed to maintain an optimal level of services for patients with limited English proficiency.

This study is limited by its cross-sectional, descriptive design and causality cannot be determined. It is also limited by the fact that patients with low literacy or lack of ease with tablets could not participate, likely underestimating needs and dissatisfaction with care. As such, ICLAP was designed for hypothesis generation for future studies with longitudinal designs, and interventions designed to elicit changes on satisfaction with care and potential alterations to the existing standard for how we are delivering care in this setting. Further, a small number of patients (11 patients) could not participate because the survey was not available in their languages (i.e. Polish, Japanese). It might be more critical to understand the levels of satisfaction of patients that speak less common languages or languages of less diffusion. Providing care for these patients might be more challenging; as such it is critically important to conduct research with patients that speak languages of lower diffusion to understand their communication needs. Additionally, while ICLAP was conducted in an urban metropolitan cancer center the patient population receiving imaging services does not always reflect the diverse urban environment of its catchment area. Finally, the results are limited to the self-report of patients and questions that our group considered to be the most appropriate to our outpatients. In our next iteration with CHIP, we will use questions (Health Information and National Trends Survey) that have been vetted in the cancer control community setting to better understand how our Center can be an active participant in cancer control and survivorship in New York City.

## Conclusion

The tenth regulation of the National Standards for Culturally and Linguistically Appropriate Services in Health Care is to conduct ongoing assessments of the organization’s CLAS-related activities and integrate CLAS-related measures into measurement and continuous quality improvement activities [16]. This assessment should include different assessments and stakeholders. The perspective of the patients should be a pivotal part of this comprehensive assessment, and the consideration of the impact of the CLAS standards on the care for patients should also be part of the assessment. In this study, a measurement tool was designed to capture patients’ perspective about the communication and language assistant services at a comprehensive cancer center. More research is needed to develop, refine, validate, and implement tools to measure the quality of care for LEP patients and the implementation of the CLAS regulations. Our long term goal is to develop and improve ICLAP, Interventional Cultural and Language Assistant Program, a comprehensive program that includes patients’ and providers’ assessments, training for providers and staff, and interventions to improve the access of language assistance and cultural competency of medical staff. We aim to expand this program to underserved community members in need of optimal preventive and cancer screening services. To this aim, we are developing a study (CHIP) to study the access to cancer care, cancer screening and communication needs to be delivered in ethnically diverse communities of New York City. This needs assessment study will use novel health technology allowing us to, first understand the needs of the community and, second to address the cancer prevention needs of ethnic and racially diverse community members.

## Figures and Tables

**Table 1:** Demographic characteristics

	n	Percentage (%)
**Age**	51.9	(18.1)
**Gender**		
Male	318	(56.4)
Female	244	(43.3)
**Education**		
Less than HS	37	(6.6)
HS graduate	57	(10.1)
Some college	105	(18.6)
College Graduate	187	(33.2)
Post-college/Graduate School	164	(29.1)
**Employment**		
Employed	253	(44.9)
Retired	121	(21.5)
Self-employed	70	(12.4)
Unable to work or Out of Work	74	(13.1)
Other	41	(7.3)
**Race/Ethnicity**		
White or Caucasian	379	(67.2)
Hispanic or Latino	76	(13.5)
Black or African American	42	(7.4)
Asian	32	(5.7)
South Asian	11	(2.0)
American Indian or Alaska Native	1	(0.2)
Other	10	(1.8)
**Birthplace**		
USA	422	(74.8)
Hispanic Caribbean-PR, Dom. Rep., Cuba	12	(2.1)
Anglo Caribbean and Haiti	16	(2.8)
Latin America, inc., Brazil	13	(2.3)
Western Europe inc., Israel and Turkey	31	(5.5)
Eastern Europe inc., Poland	20	(3.5)
Eastern Asia and Philippines	24	(4.3)
Other	24	(4.3)
**Cancer diagnosis**		
Lymphoma	98	(17.4)
Lung	67	(11.9)
Breast	46	(8.2)
Multiple Myeloma	35	(6.2)
Head and Neck	33	(5.9)
Prostate	28	(5.0)
Colorectal	27	(4.8)
Melanoma	16	(2.8)
Cervical	11	(2.0)
Other	227	(40.2)

**Table 2:** Linguistic and Interpretation-related questions

	n	Percentage (%)
Preferred language for healthcare		
English	531	(94.1)
Spanish	5	(0.9)
Mandarin/Cantonese	13	(2.3)
French/Creole	3	(0.5)
Arabic	3	(0.5)
Other	2	(0.4)
Dominant language		
English	499	(88.5)
Spanish	22	(3.9)
Mandarin/Cantonese	14	(2.5)
Russian	7	(1.2)
Arabic	4	(0.7)
Other	12	(2.1)
English proficiency		
Not at all	6	(1.0)
Not well	16	(2.8)
Well	57	(10.1)
Very well	485	(86.0)
Awareness of interpretation services	348	(61.7)
Interpretation Need	31	(5.5)
Yes, always	9	(1.6)
Yes, often	4	(0.7)
Yes, sometimes	18	(3.2)
Never	519	(92.0)
Use of interpretation services in clinic	30	(5.3)
Interpreter Type		
A friend or relative	16	(2.8)
A staff person	7	(1.2)
A trained medical interpreter	3	(0.5)
Health care provider	8	(1.4)
Frequency of not understanding doctor because of language		
Never	461	(81.7)
Always-Sometimes	96	(17.0)
Feeling comfortable discussing medical condition in a language other than English		
Never	497	(88.1)
Always-Sometimes	67	(11.9)
Hard time speaking with or understanding a provider because of language		
Never	480	(85.1)
Always-Sometimes	68	(12.1)
Understanding of what the doctor said		
Fully	495	(87.8)
Somewhat to not at all	69	(12.2)
Perception of how well the doctor understood the patient		
Completely	449	(79.6)
Mostly to not at all	103	(18.3)
Religious beliefs affect medical decisions		
Yes/Maybe	75	(13.3)
No	489	(86.7)
Ethnic or cultural background affect medical decisions		
Yes/Maybe	55	(9.8)
No	508	(90.1)
Sexual orientation affect medical decisions		
Yes/Maybe	20	(3.5)
No	544	(96.5)

Note. Because of missing data, percentages may not equal 100

**Table 3:** Unadjusted and adjusted logistic regression models predicting less satisfaction with care from service and from cancer center

	Less satisfaction with care of the service				Less satisfaction with care of the center			
	Unadjusted		Adjusted		Unadjusted		Adjusted	
Response	OR	CI	OR	CI	OR	CI	OR	CI
**Cultural factors**								
Race and ethnicity								
White or Caucasian	1.00		1.00		1.00		1.00	
Black or African American	1.08	(0.48–2.43)	1.07	(0.46–5.26)	1.25	(0.46–3.37)	1.10	(0.38–3.17)
Asian	2.40	(1.10–5.20)*	1.96	(0.84–4.59)	4.20	(1.85–9.55) ***	2.95	(1.18–7.38) *
Hispanic or Latino	0.69	(0.34–1.42)	0.54	(0.25–1.18)	0.25	(0.06–1.06)	0.09	(0.01–0.69) *
Other	1.35	(0.48–3.77)	1.29	(0.44–3.79)	2.05	(0.66–6.39)	2.16	(0.64–7.18)
Birthplace								
USA	1.00		1.00		1.00		1.00	
Latin America	0.18	(0.02–1.36)	0.23	(0.03–1.75)	0.86	(0.20–3.80)	0.92	(0.20–4.33)
Europe	0.47	(0.18–1.23)	0.53	(0.20–1.40)	0.57	(0.17–1.90)	0.65	(0.19–2.25)
Eastern Asia and Philippines	3.10	(1.33–7.24) **	2.95	(1.16–7.51) *	5.43	(2.24–13.16) ***	4.69	(1.72–12.84) **
Other	1.45	(0.68–3.08)	1.51	(0.69–3.30)	1.29	(0.48–3.48)	1.43	(0.51–3.97)
Religious beliefs affect medical decisions								
No	1.00		1.00		1.00		1.00	
Yes/Maybe	1.70	(0.97–2.99)	1.37	(0.75–2.50)	1.53	(0.76–3.09)	1.09	(0.49–2.45)
Ethnic or cultural background affect medical decisions								
No	1.00		1.00		1.00		1.00	
Yes/Maybe	2.80	(1.55–5.09) ***	2.63	(1.37–5.05) **	2.65	(1.31–5.37) **	2.23	(1.00–5.06) *
Sexual orientation affect medical decisions								
No	1.00		1.00		1.00		1.00	
Yes/Maybe	3.77	(1.52–9.35) **	2.43	(.87–6.86)	7.85	(3.11–19.82) ***	4.75	(1.62–13.96) **
**Language assistance factors**								
Preferred language for healthcare								
English	1.00		1.00		1.00		1.00	
Non-English	3.02	(1.33–6.87) **	3.49	(1.35–8.98) **	7.16	(3.04–6.85) ***	8.44	(3.02–23.57) ***
Dominant language								
English	1.00		1.00		1.00		1.00	
Non-English	1.75	(0.93–3.29)	1.76	(0.86–3.59)	2.97	(1.46–6.05) **	2.66	(1.16–6.07) *
English proficiency								
Very well	1.00		1.00		1.00		1.00	
Not at all - Well	1.33	(0.75–2.36)	1.30	(0.68–2.50)	2.15	(1.12-4-4.14) *	1.79	(0.83–3.87)
Awareness of interpretation services								
No	1.00		1.00		1.00		1.00	
Yes	1.03	(0.66–1.59)	1.04	0(.66–1.64)	1.31	(0.74–2.32)	1.42	(0.76–2.63)
Interpretation Need								
No	1.00		1.00		1.00		1.00	
Yes	3.05	(1.42–6.51) **	3.03	(1.27–7.26) **	5.94	(2.67–13.19) ***	5.49	(2.11–14.30) ***
Use of interpretation services in clinic								
No	1.00		1.00		1.00		1.00	
Yes	2.28	(1.03–5.02) *	2.04	(0.83–5.02)	4.14	(1.80–9.53) ***	3.01	(1.30–9.30) **
**Communication factors**								
Not understanding doctor because of language								
Never	1.00		1.00		1.00		1.00	
Always-Sometimes	1.78	(1.06–2.99) *	1.86	(1.05–3.29) *	3.18	(1.74–5.79) ***	3.01	(1.53–5.92) ***
Feeling comfortable discussing medical condition in a language other than English								
Never	1.00		1.00		1.00		1.00	
Always-Sometimes	2.21	(1.26–3.90) **	2.18	(1.15–4.11) *	4.04	(2.16–7.56) ***	3.68	(1.78–7.61) ***
Hard time speaking with or understanding a provider because of language								
Never	1.00		1.00		1.00		1.00	
Always-Sometimes	1.70	(0.94–3.06)	1.48	(0.76–2.87)	3.00	(1.56–5.79) ***	2.26	(1.05–4.87) *
Understanding of what the doctor said								
Fully	1.00		1.00		1.00		1.00	
Somewhat to not at all	3.94	(2.30–6.75) ***	4.40	(2.43–7.94) ***	7.40	(4.07–13.48) ***	9.05	(4.52–18.11) ***
Perception of how well the doctor understood the patient								
Completely	1.00		1.00		1.00		1.00	
Mostly to not at all	2.64	(1.62–4.30) ***	2.69	(1.60–4.53) ***	4.32	(2.40–7.76) ***	4.16	(2.20–7.86) ***

Note.

* p<0.05,

** p<0.01,

*** p<0.001;

Models adjusted for age, gender, education, employment status
